# Temporal Integration of Motion Streaks in Migraine

**DOI:** 10.3390/vision2030027

**Published:** 2018-07-13

**Authors:** Louise O’Hare

**Affiliations:** School of Psychology, University of Lincoln, Lincoln LN6 7TS, UK; LOHare@lincoln.ac.uk; Tel.: +44-1522-835720

**Keywords:** migraine, temporal integration, motion perception

## Abstract

Migraine is associated with differences in visual perception, specifically, deficits in the perception of motion. Migraine groups commonly show poorer performance (higher thresholds) on global motion tasks compared to control groups. Successful performance on a global motion task depends on several factors, including integrating signals over time. A “motion streak” task was used to investigate specifically integration over time in migraine and control groups. The motion streak effect depends on the integration of a moving point over time to create the illusion of a line, or “streak”. There was evidence of a slower optimum speed for eliciting the motion streak effect in migraine compared to control groups, suggesting temporal integration is different in migraine. In addition, performance on the motion streak task showed a relationship with headache frequency.

## 1. Introduction

Migraine is a common, debilitating neurological disorder affecting around 13% of the adult population [1]. The International Headache Society classification criteria [2] for migraine requires a minimum of five episodes of headache attacks that last 4–72 h when untreated, with the following qualities: headache is located on one side of the head, the headache has a pulsating quality, moderate to severe pain, and the headache is aggravated by physical activity. Additionally, the headache is accompanied by either nausea/vomiting, and/or by aversion to light and sound (photophobia and phonophobia, respectively). Approximately 39% of those with migraine experience migraine aura, a set of sensory disturbances preceding the onset of the headache itself [3], and the most commonly reported modality for aura is visual [1]. There is a distinction between migraine with aura (MA) and migraine without aura (MO).

Migraine has strong associations with vision: around 80% of those with migraine are light hypersensitive [4]. Those with migraine tend to report extreme discomfort on viewing striped patterns in between migraine attacks (interictally) [5]. Additionally, there are many differences in performance between interictal migraine and control groups on a variety of visual tasks (see [6], for a review). One of the most robust findings is poorer performance of interictal migraine groups compared to control groups on global motion tasks (e.g., [7,8,9,10,11,12,13]).

Global motion tasks typically involve detecting the direction of a coherently moving field of dots (or other elements) against a background of noise elements, for example dots moving in random directions. Success on a global motion task depends on multiple processes: detecting the local motion signals, then integrating the motion signals, and finally discriminating the direction of the signal from background noise [14]. The ability to detect coherent motion in the absence of stimulus noise (i.e., detecting local motion) is unimpaired in migraine compared to control groups [13]. Therefore, it appears that integration of motion and/or the ability to discriminate signal from background noise is impaired in those with migraine.

The motion streak effect depends on temporal integration: this is a visual phenomenon where a fast-moving point stimulus can be integrated over time to produce the appearance of a line in the direction of motion [15]. Streaks are also called “speedlines” and are used in artworks to imply motion in the static image [16]. The visual system can exploit this streak effect to enable the use of orientation detectors to facilitate the detection of the direction of motion ([15,17,18,19,20]). In short, a point light source moving rapidly is integrated to enable the use of orientation detectors to facilitate direction discrimination.

**Figure 1:**
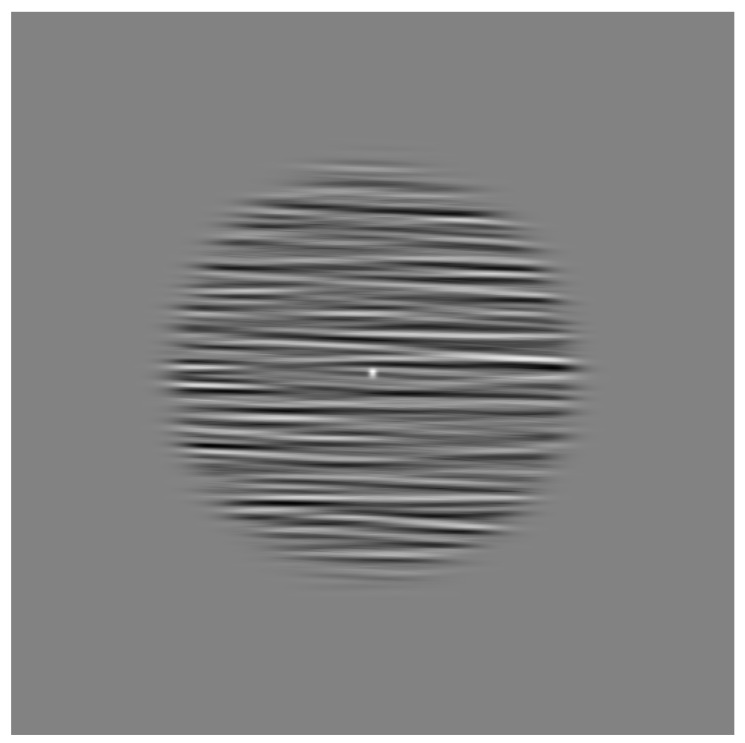
Figure showing schematic diagram of the stimulus. The Gaussian dot is presented in the centre of the screen, and moves either up, down, left or right. The task is to identify dot motion and indicate this using the corresponding arrow keys. There were five levels of dot contrast (relative to midgrey) and five possible speeds (1,4,7,10,13${}^{\circ }$/s) . The horizontal background orientation is shown. There is also a vertical background and homogenous grey background. Responses for motion orthogonal to the background were pooled, as were responses to motion parallel to the background. These were normalised against performance against the homogenous grey background.

Orientation detectors are subject to iso-feature suppression, a form of inhibition from neighbouring detectors of a similar type [21], which are sharpened by short-range inhibitory processes [22]. Therefore, the ability to use orientation detectors to discern motion direction can be estimated by comparing the masking effects of a parallel vs. orthogonal noise mask background; see Figure 1 for a schematic of the stimulus. Backgrounds oriented parallel to the motion direction will mask the streak effect, resulting in poorer performance ([15,16,17,18,19]). Additionally, backgrounds orthogonal to motion direction can facilitate the motion direction discrimination [15].

To investigate temporal integration processes, the speed of the moving element was varied, as the most effective speed for the motion streak effect is dependent on the size and contrast of the moving element. The strength of the motion streak depends on the speed of the moving element [15], the size of the moving element [15], and the distance travelled by the moving element in a given display time (number of frames) [23]. A stronger motion streak effect leads to improved ability to discriminate motion direction [15,23], and increasing the speed (up to a point) is one method of increasing the strength of the motion streak effect. There is individual variation in the critical speed for the motion streak effect (the speed at which the motion streak effect begins to appear), which will depend on the length of the temporal integration window for an observer.

Vertical and horizontal motion were estimated in the current study, as there are differences in optokinetic reflex eye movements in those with MA as well as those with migraineous vertigo, compared to control groups—those with MA show increased velocity for ocular following movements (the eyes move faster) compared to control participants [24]. Additionally, optokinetic stimulation has been demonstrated to increase motion sickness in those with migraine more than control participants [25]. The optokinetic effect is greater for horizontal compared to vertical motion in both non-human primates [26], and in humans [27]. As there is a difference in the strength of the optokinetic effect in the horizontal compared to vertical directions, and this is different in migraine and control groups, it is possible that any results could be accounted for by eye movements. To control for this possibility, it was important to estimate the effect in both planes (including all four directions of up, down, left and right).

To summarise, the motion streak effect relies specifically on temporal integration processes. Motion streak in the presence of a background oriented parallel to the motion direction will result in the greatest threshold elevation (poorest performance). This elevation in thresholds is thought to be due to orientation-specific inhibitory processes that arise due to the integration of the motion signal into a line [15]. It is expected that there will be a difference between migraine and control groups for the motion streak task, if there are differences in temporal integration processes in those with migraine. Further, it is expected that there will be differences in the optimum speed for the motion streak effect between the migraine and control groups, because the optimum speed depends on the relative speed of the motion compared to the temporal integration window.

## 2. Results

### 2.1. Orthogonal Motion

**Figure 2:**
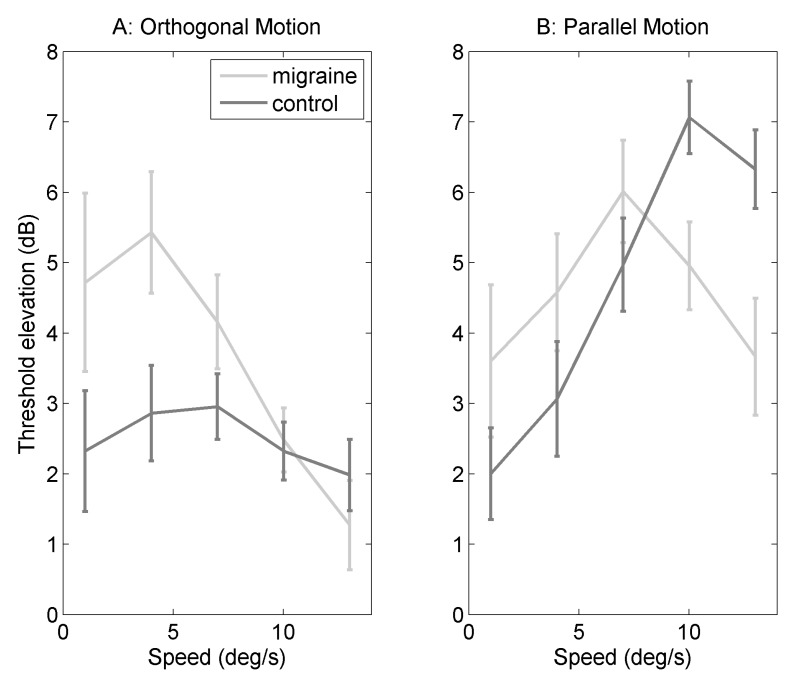
Figure showing threshold elevation (dB) against speed of dot motion for migraine and control groups. Error bars are one standard error of the mean: (**A**) threshold (dB) against speed (deg/s) for motion orthogonal to the background orientation; and (**B**) threshold (dB) against speed (deg/s) for motion parallel to the background orientation.

Figure 2A shows threshold elevation for five speeds of dot motion (1, 4, 7, 10 and 13${}^{\circ }$/s) against orthogonal noise backgrounds (elevation compared to homogenous grey background). Mixed ANOVA was conducted with group as a between subjects factor, and speed as a within-subjects factor, degrees of freedom were adjusted where the assumption of homogeneity of variance was not met (see Section 4 for more details). There was a significant main effect of speed (F(2.95,144.57) = 4.23, *p* = 0.007, $\eta_{G}^{2}$ = 0.06). Post-hoc least-squares means contrast were calculated using the package “lsmeans” (for details, see Section 4 [28]), using the Bonferroni correction for ten comparisons. There was a significant difference between 4 and 13${}^{\circ }$/s (*p* = 0.0040), none of the other comparisons were statistically significant after correction for multiple comparisons. Threshold elevation was overall greater for the migraine group compared to the control group (F(1,49) = 5.70, *p* = 0.02, $\eta_{G}^{2}$ = 0.03). There was no statistically significant interaction between group and speed (F(2.95,144.57) = 2.05, *p* = 0.11, $\eta_{G}^{2}$ = 0.03).

### 2.2. Parallel Motion

Figure 2B shows threshold elevation (dB) for motion against parallel filtered noise backgrounds (elevation was calculated by dividing threshold against filtered noise background by threshold for homogenous grey background). Mixed ANOVA was conducted with group as a between-subjects factor, and speed as a within-subjects factor (see Section 4 for more details). There is a significant main effect of speed (F(3.08,150.86) = 6.33, *p* = 0.0004, $\eta_{G}^{2}$ = 0.09). There is no effect of group (F(1,49) = 0.07, *p*< 0.80, $\eta_{G}^{2}$ = 0.0003). There is an interaction between migraine and speed (F(3.08,150.86) = 4.06, *p* = 0.008, $\eta_{G}^{2}$ = 0.06). There was a significant difference between migraine and control groups at 10${}^{\circ }$/s (*p* = 0.044) and at 13${}^{\circ }$/s (*p* = 0.011), none of the other comparisons were statistically significant when adjusting for multiple comparisons using Bonferroni correction.

### 2.3. Difference in Threshold Elevation

**Figure 3:**
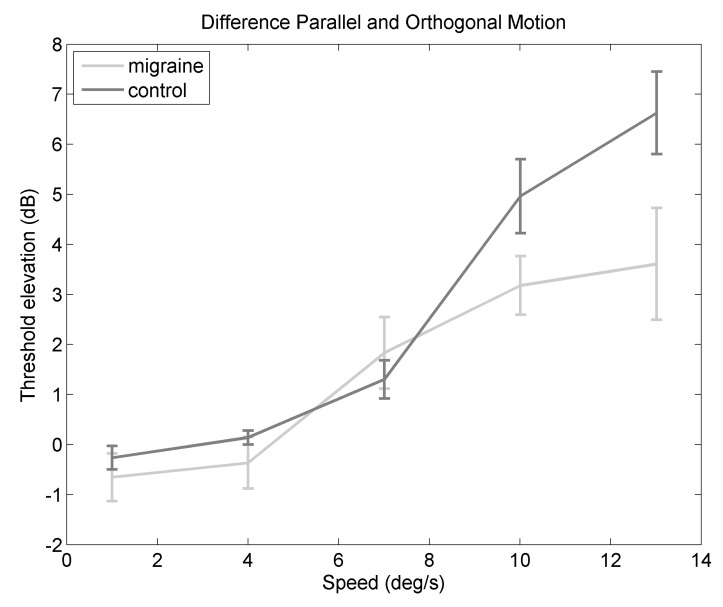
Figure showing difference in threshold elevation (parallel-orthogonal background) against speed for migraine and control groups. Error bars are one standard error of the mean.

Figure 3 shows the *difference* in threshold elevation between orthogonal and parallel backgrounds, to highlight the cost of the parallel background compared to the orthogonal background. There is a significant main effect of speed (F(2.89,141.78) = 34.04, *p* < 0.0001, $\eta_{G}^{2}$ = 0.34). Post-hoc comparisons showed a significant difference between 1 and 7${}^{\circ }$/s (*p* = 0.0093), between 1 and 10${}^{\circ }$/s (*p* < 0.0001), and between 1 and 13${}^{\circ }$/s (*p* < 0.0001). There was also a significant difference between 4 and 10${}^{\circ }$/s (*p* < 0.0001), and between 4 and 13${}^{\circ }$/s (*p*< 0.0001). There was a significant difference between 7 and 10${}^{\circ }$/s (*p* = 0.0005), and between 7 and 13${}^{\circ }$/s (*p* < 0.0001). The other comparisons were not statistically significant when adjusting fo multiple comparisons using the Bonferroni correction. This indicates that the increase in masking between orthogonal and parallel backgrounds depends on speed. The difference between orthogonal and parallel backgrounds is smaller for the migraine group compared to the control group (F(1,49) = 5.40, *p* = 0.02, $\eta_{G}^{2}$ = 0.03). The interaction between speed and migraine group is not statistically significant (F(2.89,141.78) = 2.63, *p* = 0.05, $\eta_{G}^{2}$ = 0.04), however the effect size estimate indicates a small effect.

### 2.4. Relationship to Headache Frequency

Frequency of headaches was assessed using a questionnaire. Participants responded by choosing one of the following options: whether their headaches occurred never, less than once a month, 1–3 times a month, 3–10 times a month, or more than 10 times a month. Estimated frequency of headaches was coded to take the midpoint of each category of answers, for example, 1–3 times a month was recoded as 2, the midpoint. Values less than 1 were coded as 0.5, values of 10 or more were coded as 10. A linear mixed effects model was fitted to see if headache frequency of the migraine group predicted the difference in threshold elevation (orthogonal minus parallel, as in Figure 3). The linear mixed effect model has some advantages in terms of non-independent observations, for a discussion, see [29]. An alternative model included headache frequency and dot speed as fixed factors, and also observer as a random factor. The null model included only the factors of dot speed as a fixed factor, and observer as a random factor. Thus, two models were compared: the alternative model includes headache frequency, and the null model does not include headache frequency as a predictor. The ability to predict the difference in threshold elevation was significantly better for the alternative model (with headache frequency) compared to the null model (without headache frequency) ($\chi^{2}$ (1) = 8.01, *p* = 0.0046). The difference in threshold elevation motion against orthogonal and parallel backgrounds increased with headache frequency by 0.32 dB (standard error = 0.11). This indicates a greater threshold elevation with those who experience more frequent headaches.

## 3. Discussion

The motion streak task specifically was chosen as this effect depends on temporal integration processes [15]. Importantly, backgrounds orthogonal to motion direction should present less masking than backgrounds parallel to motion direction [15]. For both groups, the experiment partially replicated the motion streak effect originally found by Geisler [15]—a detrimental effect of the background oriented parallel to the motion direction, which can be seen in Figure 2B.

Importantly, there are group differences shown on the motion streak task; those with migraine showed poorer performance for orthogonal backgrounds. For motion against parallel backgrounds, there is an interaction between migraine and speed, with a bigger motion streak effect in the control group compared to the migraine group at faster speeds. In Figure 2B, the migraine group reach asymptote at slower speeds compared to the control group. This suggests the optimum speed for the motion streak is slower for the migraine compared to the control group. To highlight the motion streak effect, threshold elevation against orthogonal and parallel backgrounds was compared, a greater difference would be evidence of a greater motion streak effect, which can be seen in Figure 3. Figure 3 shows a lower difference between motion parallel and orthogonal to the background for the migraine group compared to the control group, indicating a weaker motion streak effect. Therefore, it may be the case that there is a shorter temporal integration window in migraine, as the streak effect occurs earlier, and is weaker, in this group.

The motion streak effect (difference in threshold elevation for motion against orthogonal backgrounds compared to motion against parallel backgrounds) was estimated in both vertical and horizontal motion direction. Both motion orientations were used to control for the possibility of anisotropy in performance, as there is evidence that the optokinetic effect is different in migraine compared to control groupss [24,25]. The motion streak effect was found in both horizontal and vertical motion directions (see Appendix B), and so both directions of motion (set against orthogonal and parallel backgrounds) were used in the main study. When direction of motion (horizontal and vertical dot motion) was included as a factor in the analysis of the difference in threshold elevation between orthogonal and parallel backgrounds, there was some evidence of poorer performance overall for the vertical motion direction compared to the horizontal motion direction, but no group-specific effect of direction of motion. Therefore, the poorer performance for vertical compared to horizontal dot motion may be evidence of the optokinetic effect, but this appears to be no different in the migraine and control groups in the current study.

### 3.1. Relationship to Migraine Characteristics

Headache frequency (in the migraine group) was predictive of a greater difference in threshold elevation for motion against the orthogonal compared to the parallel backgrounds. Those with more frequent headaches tend to show a bigger difference, and so bigger motion streak effects. A relationship between attack frequency and cognitive test performance, including tasks of visual memory has been shown previously (e.g., [30]). Additionally, those with migraine show elevated response amplitude when measured over the somatosensory cortex, which is positively correlated to the frequency of migraine attacks [31]. However, the relationship between headache frequency and cognitive test performance is mixed (see [32] for a review). Visual field deficits were not found to correlate with attack frequency [33,34].

The current study only considers interictal migraine—those who had experienced a migraine attack too recently were excluded from the study. Effects of the migraine cycle would be interesting to investigate in future research, as there are effects of the migraine cycle shown in both behavioural (e.g., [35,36]) and electrophysiological [37,38,39] responses. Due to multiple testing sessions it was not possible to estimate effects of the migraine cycle in the current experiment. The use of a staircase procedure would enable thresholds to be obtained more rapidly (therefore no longer needing multiple testing sessions) and also avoid potential problems with floor effects.

### 3.2. Temporal Integration Mechanisms

The integration of two (or more) rapidly presented stimuli is of particular importance in migraine, as those with migraine report aversion on viewing flickering stimuli (e.g., [40,41]). It has been suggested that flickering stimuli, such as the “two flash” test is a simple estimate of temporal integration, without the spatial component that is inherent in motion perception [20] . The critical flicker fusion threshold is the lowest frequency at which flickering light will be seen as detectable, rather than a steady light source. Previous studies reported lower flicker fusion thresholds in migraine compared to control groups [42,43], which would be indicative of increased summation of responses over time. In the case of the work of Coleston and Kennard [42], there is a significant difference in the flicker fusion threshold between MO and control groups, but not between MA and control groups. This is not in agreement with findings that those with migraine are better able to detect briefly presented stimuli compared to control group [44], or the current study showing a weaker motion streak effect in the migraine group. It is unclear why this might be the case. Speculatively, it has been shown that habituation to repetitive stimuli is weaker in migraine compared to control group, indicating that there is a sustained response to repetitive stimuli in the migraine group [45]. This could be an important difference between temporal integration of single stimuli compared to repetitive stimulation.

Brain connectivity is important for the integration of sensory information, and this has been suggested to be a potential cause of sensory processing differences seen in those with migraine [46]. Local functional connectivity within visual areas is unimpaired in those with migraine [46]. Further, connectivity in the resting state has been shown to be increased in those with MA compared to control groups [47]. Temporal integration of multisensory stimuli has been linked to functional connectivity in EEG studies [48,49]. Functional connectivity in terms of coherent neural oscillations has been shown to be different in those with migraine compared to control groups [50]; those with MA showed stronger beta band coherence compared to MO and control groups, whereas those with MO showed weaker alpha band (8–12 Hz) coherence compared to MA and control group. Alpha band (8–12 Hz) oscillations in the occipital areas of the brain are thought to control the timing of information processing in the visual areas of the brain [51]. The alpha band oscillations provide a “window of excitability” [52]. If alpha power is high at stimulus onset, then it is unlikely to be perceived; conversely, if occipital alpha power is low at stimulus onset, then it is more likely to be perceived [53,54,55]. Increasing alpha band frequency has been demonstrated to have effects on the temporal integration window in normal observers for multisensory integration studies [56,57]. Evidence for a longer integration window has been demonstrated in multimodal integration between the visual and vestibular system in those with migraine [58]. The current study shows some evidence that the temporal integration window is different in migraine compared to control groups, and this task is thought to depend on integration of the signal over time in order for the streak to appear. In particular, there is evidence of a slower optimum speed for the migraine group compared to the control group, and so it might be thought that the neural oscillations may also be slower in those with migraine compared to control groups. In future research, it would be useful to assess whether temporal integration relates to coherence of neural oscillations (functional connectivity) in those with migraine, as this has clear predictions for clinical outcomes and potential treatments [59].

### 3.3. Limitations of the Current Study

In the current experiment, any potential group differences in motion detection, or lateral inhibition *per se* were controlled for by considering threshold elevation between oriented backgrounds and homogenous grey backgrounds. Previous research has shown increased lateral inhibition in migraine compared to control group using behavioural measures [60]. Additionally, electrophysiological data have also shown differences in short-range lateral inhibition in those with migraine [61]. However, there is evidence [44,62] that the short-range lateral interactions are no different in migraine compared to control groups. Importantly, if there were a difference in short-range interactions, both vertical and horizontal background conditions should be equally affected, which was not the case. Additionally, if the migraine group is simply more susceptible to noise from the addition of an oriented background compared to control group, then this should affect both backgrounds (orthogonal and parallel to the direction of motion) equally, and so no specific motion streak effect should be seen. Again, this was not the case. It is also possible that differences in the ability to detect motion in migraine—for example, differences in the opponent motion system [63]. There is increased cortical thickness in area V3A and area MT+, both of which are associated with motion processing [64], which might be expected to result in potential differences in motion processing in migraine compared to control groups. However, there is evidence to suggest that the detection of motion in the absence of noise is unimpaired in migraine groups [13]. Even so, the difference in threshold elevation between the parallel and orthogonal the background was estimated to limit the effect of differences in motion detection between the two groups accounting for the results in the current experiment.

One limitation of the current study is that not all individuals had a diagnosis of migraine by a medical professional. It has been suggested that many of those with migraine do not seek medical advice, estimates vary between 37% [65] and around 50% [66]. Participants in the current study were asked to complete a questionnaire to assess whether they fulfilled the International Headache Society criteria [2], diagnosis was not made by a trained neurologist. To address this concern, Appendix C contains a second analysis of the results, including only those individuals with a diagnosis of migraine from a medical professional. There is a similar pattern of results when only these individuals are included in the migraine group. A review of cognitive deficits in migraine showed that migraine with aura was inconsistently associated with cognitive deficits, including performance on visually-based tasks, in migraine [32]. Unfortunately, in this study, there were insufficient participants to be able to split the migraine group into MA and MO groups. This is a limitation of the study, as it is not clear if these two groups differ in terms of their performance on this task. Those with MA do show differences to those with MO (e.g., [50]), but this is not always the case, as other studies have not shown differences in motion perception between MA and MO groups (e.g., [8,9,13,67,68]).

Another limitation is the reliance on self-report for the measures of normal visual acuity. It is possible that individuals have uncorrected visual deficits that they may or not be aware of. It has been shown previously that differences in contrast sensitivity also can correlate with performance on motion based tasks in those with migraine e.g., [12]. Poorer visual acuity and/or differences in contrast sensitivity could account for the overall poorer performance in the migraine group compared to the control group. However, the difference in threshold elevation cannot be accounted for by poorer acuity or reduced contrast sensitivity, as either of these would result in the same decrease in both conditions, not an orientation-specific effect of the background.

Recent fMRI studies demonstrate that brain networks associated with attention have been shown to be different in those with migraine compared to control group in between migraine attacks (e.g., [69]). Additionally, Coppola et al. [70] showed reduced thalamocortical connectivity in MO compared to control group at resting state during a migraine attack. Other researchers have also demonstrated reduced activity at rest in networks associated with attention in MO, but not MA, compared to control groups [71]. If attention is different in those with migraine compared to control group, then this might be expected to account for differences in susceptibility to externally added noise. Differences in attentional network connectivity could account for the overall poorer performance in the migraine group compared to the control group in the current study. However, differences in attention alone cannot account for the difference in threshold elevation between the orthogonal and parallel backgrounds.

## 4. Materials and Methods

### 4.1. Observers

Raw data for the experiment can be found in the Supplementary Materials. Sixty observers with corrected-to-normal vision were recruited to the study using advertising around the university campus. Visual acuity was based on self-report; if participants reported needing optical corrections, they were encouraged to wear them. All experiments adhered to the World Medical Association Declaration of Helsinki (2013) and were scrutinised by the University of Lincoln School of Psychology Ethics committee, ethical approval code PSY1617240. Written informed consent was obtained from all observers before commencing the study.

Migraine was defined as fulfilling the International Headache Society diagnostic criteria [2]. This was determined via a questionnaire, administered by the experimenter. Not all individuals had a migraine diagnosis from a medical professional (e.g., GP or neurologist), but many did. The diagnosis by a medical professional can be seen in Table 1. Individuals with “probable migraine”, who experience an insufficient absolute number of headache attacks (fewer than five attacks) were excluded. Five observers did not fulfill the criteria. Two of these five who were excluded as they did not fulfill the IHS criteria had a diagnosis of migraine by a medical professional. These were excluded from the analysis here, but included in the analysis of only those with a professional diagnosis of migraine in the Appendix C. Two additional observers were excluded as they had a diagnosis of tension headache, and chronic headache, respectively. Those with a headache attack within two days were excluded (one observer). Control groups contained individuals who had reported no migraine, and no regular headaches. One control participant was excluded due to experiencing too many headaches. As the migraine classification was based on self-report, the experimenter would have known which participants were in the migraine and which were in the control group. To minimise subjective bias from this experimenter knowledge, all participants completed all conditions of the experiments, and all trials were presented in random order, by computer, therefore minimising subjective bias. After exclusions there were 29 control participants (mean age 23.517 years, SD = 6.399), eight of whom were male, and 22 migraine participants (mean age 24.82 years, SD = 8.16), three of whom were male.

### 4.2. Apparatus

All experiments took place in a sound-attenuated, darkened room. An MSI (MS-7788) computer with i7-3990CPU Intel processor, NVida GeForce GTX 650 graphics card, and a 64-bit Windows 7 operating system was used to create and display stimuli. Stimuli were displayed using a 22-inch Illyama HM204DTA Vision Master Pro 514 Diamondtron U3-CRT monitor, calibrated with LS100 Minolta photometer. A Bits Sharp signal processor (Cambridge Research Systems, Cambridge, UK) was used to convert the RGB signal into 14-bit greyscale. Mean luminance of the display used for the behavioural task was 44.24 cd/m${}^{2}$. Refresh rate was 85 Hz, resolution was 1024 × 768 pixels. Viewing distance was 100 cm, head movements were restricted using a chinrest. Matlab Version 2015a (The Mathworks, Natick, MA, USA) and the Psychtoolbox ([72,73,74]) were used to generate and display stimuli.

### 4.3. Stimuli

The stimulus was a Gaussian blob (0.1${}^{\circ }$, or 6 arcmin, of visual angle, measured across two standard deviations). Dot speed was 1, 4, 7, 10, and 13${}^{\circ }$/s. The dot always began in the centre of the screen. The dot could move either horizontally or vertically. There were again five levels of dot contrast, between 0% and 10% contrast, compared to the homogenous mid-grey background. There were three background types, no background (homogeneous grey), filtered noise oriented parallel to the motion, and filtered noise oriented perpendicular to the motion. The filter was a raised radial cosine function, see Equation (1).

(1) $$\begin{array}{cc} H(f) & =\left\{\begin{array}{c} T\\ \frac{T}{2}[1+cos\left(\frac{\pi T}{\beta }\right)(|log\left(f\right)-log\left(f_{0}\right)|-\frac{1-\beta }{2T})]\\ O \end{array}\right.\\ & \mathrm{for}\left\{\begin{array}{c} \left(0\leq |log\left(f\right)-log\left(f_{0}\right)|\leq \frac{1-\beta }{2T}\right)\\ \left(\frac{1-\beta }{2T}\leq |log\left(f\right)-log\left(f_{0}\right)|\leq \frac{1+\beta }{2T}\right)\\ \left(|log\left(f\right)-log\left(f_{0}\right)|>\frac{1+\beta }{2T}\right) \end{array}\right. \end{array}$$

where *T* is 0.9, beta is 0.5, and *f*${}_{0}$ is the centre frequency is of 2.9 cycles/${}^{\circ }$. The bandwidth of the filtered noise was relative to the dot size, the centre frequency ± 0.1${}^{\circ }$ from the centre frequency. Stimuli were presented in a Gaussian-edged window with an aperture of 5.06${}^{\circ }$ and a soft edge σ = 0.51${}^{\circ }$. A schematic stimulus can be seen in Figure 1. There were 3 backgrounds × 5 contrast levels × 5 speeds × 20 repetitions of each trial resulting in a total of 1500 trials. Trial order was randomised for each participant individually. Observers indicated the direction of motion in a four-alternative forced-choice procedure, chosing either left, right, up or down on any given trial, by pressing the corresponding arrow keys. The experiment was divided into five blocks (300 trials in each block), over three sessions. Each session lasted around 1 h. The first block was used as a practice session and excluded from the analysis.

Stimulus parameters were chosen based on pilot work, the first pilot study was to estimate the dot size and contrast levels needed for the main experiment, using two dot sizes and horizontal motion only. Results from this can be seen in Appendix A. The second pilot study was to ensure that the stimulus parameters from the first pilot study were suitable for both horizontal and vertical motion directions. Results from this can be seen in Appendix B.

### 4.4. Statistical Analysis

The level of dot contrast needed to achieve 75% correct performance was estimated for each of the conditions. Threshold performance (against filtered noise background) was normalised to the baseline (homogenous grey background) performance to assess the effect of motion streaks, and not just performance against a mask. Normalisation was achieved by dividing the oriented background (horizontal or vertical) by the homogenous grey background. Normalised thresholds are expressed in dB. This was achieved using the following equation:

(2) $$20log_{10}\left(thresholdOriented/thresholdHomogenousGrey\right)$$

where thresholdOriented is the threshold obtained for motion against the oriented background, and thresholdHomogenousGrey is the threshold obtained against the homogenous grey background. Inferential statistics were calculated using R [75] with packages “afex”, [76], “lsmeans” [28], and “lme4” [77]. Mixed ANOVA was conducted with migraine as a between subjects factor, and background and speed as within-subjects factors. Data were not normally distributed according to a Shapiro-pWilk test: for motion against orthogonal backgrounds (W = 0.90, *p* = 6.86 $\times 10^{-12}$), for motion against parallel backgrounds (W = 0.94, *p* = 2.16$\times 10^{-8}$), and for difference in threshold elevation (W = 0.87, *p* = 8.14 $\times 10^{-14}$). However, the ANOVA can to be robust to certain violations of the assumption of normality, depending on the severity of the violation [78,79,80]. Violations of these assumptions also applies to the post-hoc tests. For completeness, non-parametric versions of the tests were performed using the R package “nparLD” [81], using the Wald test statistic, and results showed that there were no differences in the overall pattern of results between the non-parametric versions of the analysis.

## 5. Conclusions

The motion streak task was used as an estimate of temporal integration in migraine and control groups. Those with migraine showed poorer performance overall, suggesting an inability to exclude external noise from the background in migraine. For motion against parallel backgrounds, there was a more pronounced effect of dot speed for the control group compared to the migraine group, and, importantly, evidence of motion streak effects at slower speeds for the migraine group. This suggests a difference in temporal integration in the migraine compared to the control group.

## Figures and Tables

**Table 1:** Table showing characteristics of those individuals with migraine included in the final analysis, after exclusions. Monthly freq, monthly frequency of headaches; Visual, are there any visual disturbances during the attack; Speech, are there any speech disturbances during the attack; Prof diagnosis, do you have a diagnosis of migraine, migraine with aura, migraine without aura by a medical professional (either GP or neurologist); M, migraine; MO, migraine without aura; MA, migraine with aura; AB, abdominal migraine.

Gender	Age	Monthly Freq	Visual	Speech	Prof Diagnosis	Last Attack
F	28	<1	yes	yes	MA	2 weeks
M	41	<1	yes	no	no	2 months
F	23	1 to 3	yes	no	M	8 days
F	48	<1	yes	no	MA	18 months
M	19	3 to 10	yes	no	MA	2 weeks
F	29	<1	yes	yes	MA	1 month
F	18	<1	yes	no	MA	4 months
F	22	1 to 3	no	yes	no	2 weeks
F	19	3 to 10	yes	no	M	5–6 days
F	21	1 to 3	yes	no	MO	2–3 weeks
F	21	10+	yes	yes	MA	3–4 days
F	22	<1	yes	yes	AB	months
F	19	1 to 3	no	no	M	6 days
F	20	1 to 3	yes	no	M	1 week
F	21	3 to 10	yes	no	no	1 week
F	18	1 to 3	yes	no	MA	2 weeks
F	22	1 to 3	yes	no	M	6 days
F	23	1 to 3	yes	no	no	3 weeks
F	21	3 to 10	yes	no	MO	2 days
F	26	1 to 3	yes	no	M	2 weeks
M	24	<1	yes	no	no	2 months
F	41	<1	no	no	M	2 weeks

**Figure A1:**
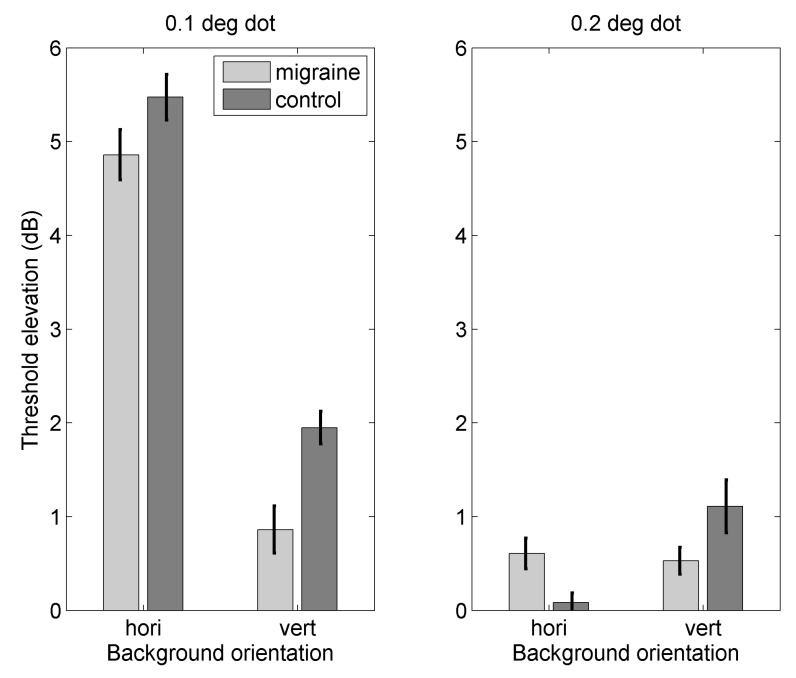
Figure showing threshold elevation for (**left**) 0.1${}^{\circ }$ dot size and (**right**) 0.2${}^{\circ }$ dot size for migraine and control groups against horizontal and vertical backgrounds. Dot motion is horizontal. Error bars are one standard error of the mean.

**Figure A2:**
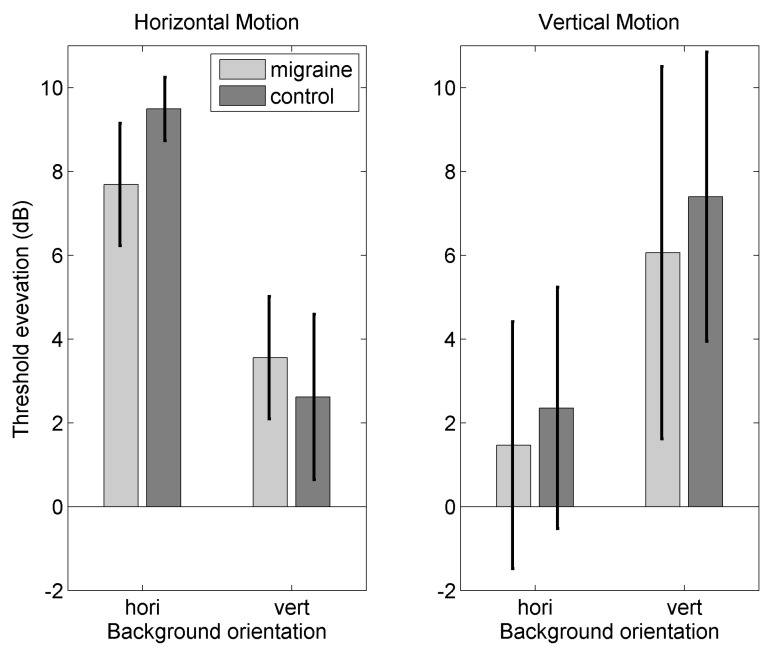
Figure showing threshold elevation for (**left**) horizontal motion and (**right**) vertical motion against vertical and horizontal backgrounds. Dot size is 0.1${}^{\circ }$. Error bars are one standard error of the mean.

**Figure A3:**
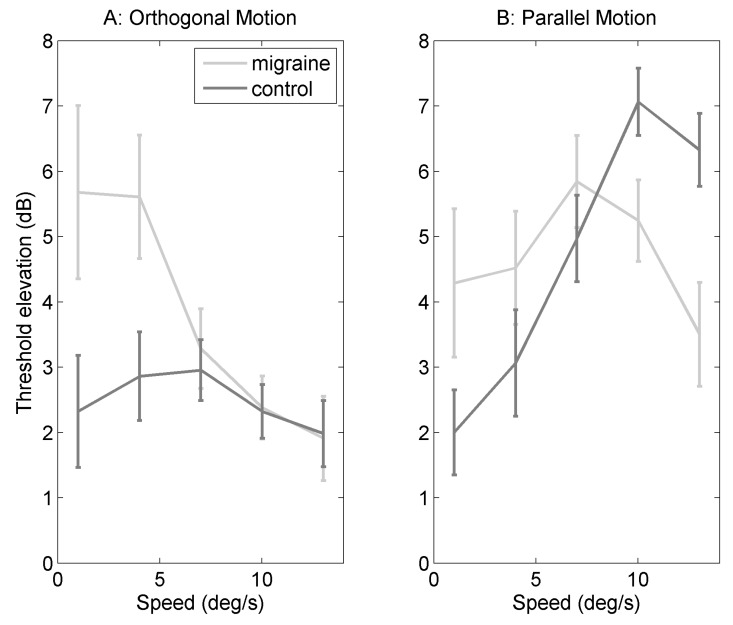
Figure showing difference in threshold elevation (parallel-orthogonal background) against speed for migraine and control groups. Error bars are one standard error of the mean.

**Figure A4:**
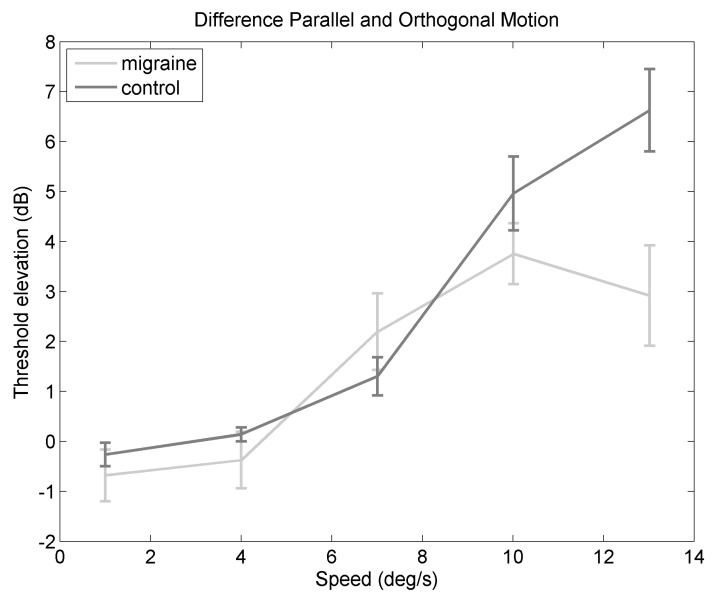
Figure showing difference in threshold elevation (parallel-orthogonal background) against speed for migraine and control groups. Error bars are one standard error of the mean.

## Supplementary Materials

Supplementary materials can be found at http://www.mdpi.com/2411-5150/2/3/27/s1.

## Abbreviations

The following abbreviations are used in this manuscript:

IHS	International Headache Society
M	migraine
MA	migraine with aura
MO	migraine without aura
tACS	transcranial alternating current stimulation

## Appendix A. Experiment 1

Experiment 1 was to determine the stimulus parameters for the main study. Previous work on the motion streak has been conducted with limited numbers of experienced psychophysical observers (e.g., [15]), and so their threshold performance may be different to the target group for this experiment. Experiment 1 consisted of only one motion speed, but two dot sizes, to test that the motion streak occurred in both those with migraine and control group, using these stimulus parameters.

### Appendix A.1. Method

#### Appendix A.1.1. Observers

Raw data for the experiment can be found in the Supplementary Materials. Fifty-two participants with corrected-to-normal vision (based on self-report only) took part in the study. Migraine was defined as fulfilling the IHS criteria (2013), or had a diagnosis from a medical professional. Individuals with “probable migraine” (insufficient frequency of headache attacks (fewer than five attacks) were excluded. One observer was excluded due to being diagnosed with cluster headache, rather than migraine. Two observers were excluded due to previous head trauma. Five further observers were excluded as their most recent headache attack was within two days. A final three observers had outlying thresholds (defined as exceeding two standard deviations of the mean), and so were excluded. Fifteen migraine and 20 control participants were included in analysis, after exclusions; see Table A1 below for migraine characteristics. The migraine group were an average age of 21.20 years (standard deviation 5.00), and two were male. The control group were an average age of 20.7 (standard deviation 2.45) years, and six of them were male.

**Table A1:** Individuals with migraine included in the first pilot study, after exclusions. These individuals are not the same group of people as those who took part in the main experiment. Monthly freq, monthly frequency of headaches; Visual, are there any visual disturbances during the attack; Speech = are there any speech disturbances during the attack; Prof diagnosis, do you have a diagnosis of migraine from a medial professional (e.g., GP or neurologist); M, migraine; MO, migraine without aura; MA, migraine with aura.

Gender	Age	Monthly Freq	Visual	Speech	Prof Diagnosis	Last Attack
F	19	1–3	no	no	M	1 month
F	18	10+	yes	no	MA	2 months
F	19	0	yes	no	M	1 week
F	19	1–3	yes	no	MA	1 week
F	20	1–3	yes	no	no	2 weeks
F	15	1–3	yes	no	M	1 week
F	18	<1	yes	no	MA	3 months
M	19	3–10	yes	no	no	2 weeks
F	19	<1	yes	no	no	2 days
F	20	0	yes	no	MA	2 weeks
F	18	3–10	no	no	no	2 months
F	32	1–3	no	no	MO	1 month
M	24	1–3	no	no	no	1 week
F	31	<1	yes	no	MA	12 months
F	27	<1	yes	yes	MA	6 weeks

#### Appendix A.1.2. Stimuli

There were two sizes of Gaussian dot used, 0.1${}^{\circ }$ and 0.2${}^{\circ }$. Dot speed was 13.02${}^{\circ }$/s There were five levels of contrast or the Gaussian blob, varying from 0% to 2.5% compared to midgrey. There were 600 trials in total, 300 trials for each dot size.

### Appendix A.2. Results

Figure A1 shows threshold elevation for migraine and control groups for two dot sizes, 0.1${}^{\circ }$ and 0.2${}^{\circ }$. Note the difference in scale. For the smaller dot size, an effect of background (F(1,33) = 27.95, *p* < 0.0001, $\eta_{G}^{2}$ = 0.18), but for the larger dot size there was no effect of background (F(1,33) = 0.55, *p* = 0.46, $\eta_{G}^{2}$ = 0.005). This is due to ceiling effects with the larger dot size, as threshold elevation was very low.

## Appendix B. Experiment 2

Raw data for the experiment can be found in the Supplementary Materials. Experiment 2 manipulated the direction of dot motion, between horizontal and vertical. This experiment was to check that the motion streak effect seen in Experiment 1 could be replicated, that it is not specific to one direction, and that the 4AFC design is suitable. 4AFC has the advantage over 2AFC as the likelihood of guessing correctly on any individual trial is reduced. There have been reports of anisotropies in motion discrimination (e.g., [82,83]), and so the inclusion of the vertical direction of motion is to see that the motion streak can also be elicited in this direction with the same parameters.

### Appendix B.1. Method

#### Appendix B.1.1. Observers

Forty-two participants with corrected-to-normal vision (based on self-report only) took part in the study, again migraine was defined as fulfilling the IHS (2013) classification criteria. One observer was excluded as they had cluster headache, rather than migraine. One observer was excluded as their migraines are the result of a diagnosed brain disorder (perniculous anemia). One observer was excluded due to reporting a headache within two days of the testing session. Four observers in the migraine group, and one in the control group, were excluded as thresholds could not be fitted to their data, due to floor effects. Ten migraine and 16 control participants were included in the final analysis, after exclusions (see Table A2 below for details). The average age of the migraine group was 19.60, years (standard deviation 1.51), and one member of the migraine group was male. There were 16 individuals in the control group, mean age was 20.75 (standard deviation 2.91), and six were male.

**Table A2:** Individuals with migraine who took part in the second pilot experiment, after exclusions. These individuals are not the same group of people who took part in the main experiment, or the first pilot experiment. Monthly freq, monthly frequency of headaches; Visual, are there any visual disturbances during the attack; Speech, are there any speech disturbances during the attack; Prof diagnosis, do you have a diagnosis of migraine by a medical professional (e.g., GP or neurologist); M, migraine; MO, migraine without aura; MA, migraine with aura.

Gender	Age	Monthly Freq	Visual	Speech	Prof Diagnosis	Last Attack
F	21	3–10	yes	no	M	1 week
F	18	3–10	yes	no	M	2 days
F	19	1–3	no	no	M	5 days
F	19	3–10	yes	no	no	3 days
F	19	<1	no	no	no	2 days
F	20	1–3	no	no	no	2 weeks
F	18	1–3	no	no	no	1 week
F	20	3–10	yes	no	M	1 day
F	19	1–3	yes	no	MA	1 month
M	23	1–3	yes	no	no	2 weeks

#### Appendix B.1.2. Stimuli

The task was 4AFC, the direction of motion could be up, down, left and right, observers indicated the direction of motion using the corresponding arrow keys. Contrast of the dot was increased slightly from Experiment 1, to five levels of contrast between 0% and 10 % compared to midgrey. There were again 600 trials in total, this time 300 trials for each direction of motion.

### Appendix B.2. Results

Figure A2 shows threshold elevation for migraine and control groups for horizontal and vertical motion. There was an effect of background for both horizontal (F(1,24) = 21.88, *p* < 0.0001, $\eta_{G}^{2}$ = 0.17) and vertical backgrounds (F(1,24) = 36.27, *p* < 0.0001, $\eta_{G}^{2}$ = 0.20). The motion streak effect is not specific to horizontal motion and so both motion directions were used in the full study.

## Appendix C. Diagnosis of Migraine by a Medical Professional Only

The analysis was repeated using only those individuals with a professional diagnosis of migraine. There were 20 individuals with a professional diagnosis of migraine, mean age was 23.90 years (standard deviation 7.76), and two of this group were male. There were 29 control group, mean age 23.52 years (standard deviation 6.39), and eight of this group were male. It is important to note that many of these are the same individuals as in the main experiment; for clarity, the individuals included in this re-analysis are listed in Table A3. There were some cases where participants were not included in the original analysis (as they did not meet the IHS criteria) but have a professional diagnosis of migraine.

**Table A3:** Individuals with a professional diagnosis of migraine only. These data are from the main experiment, this time excluding those individuals who did not have a diagnosis of migraine by a medical professional. Monthly freq, monthly frequency of headaches; Visual, are there any visual disturbances during the attack; Speech, are there any speech disturbances during the attack; Prof diagnosis, do you have a diagnosis of migraine, migraine with aura, migraine without aura by a medical professional (either GP or neurologist); M, migraine; MO, migraine without aura; MA, migraine with aura; VM, vestibular migraine; AB, abdominal migriane.

Gender	Age	Monthly Freq	Visual	Speech	Prof Diagnosis	Last Attack
F	28	<1	yes	yes	MA	2 weeks
F	23	1 to 3	yes	no	M	8 days
F	48	<1	yes	no	MA	18 months
M	19	3 to 10	yes	no	MA	2 weeks
F	29	<1	yes	yes	MA	1 month
F	18	<1	yes	no	MA	4 months
F	19	0	yes	no	VM	8–10 days
F	19	3 to 10	yes	no	M	5–6 days
M	23	0	yes	yes	M	7–9 months
F	21	<1	yes	no	M	1 month
F	21	1 to 3	yes	no	MO	2–3 weeks
F	21	10+	yes	yes	MA	3–4 days
F	22	<1	yes	yes	AB	months
F	19	1 to 3	no	no	M	6 days
F	20	1 to 3	yes	no	M	1 week
F	18	1 to 3	yes	no	MA	2 weeks
F	22	1 to 3	yes	no	M	6 days
F	21	3 to 10	yes	no	MO	2 days
F	26	1 to 3	yes	no	M	2 weeks
F	41	<1	no	no	M	2 weeks

### Appendix C.1. Threshold Elevation for Motion Against Orthogonal Backgrounds

There was a significant main effect of group (F(1,47) = 7.09, *p* = 0.01, $\eta_{G}^{2}$ = 0.03). There was a significant main effect of speed (F(2.95,138.53) = 3.96, *p* = 0.01, $\eta_{G}^{2}$ = 0.06). Post-hoc tests with Bonferroni correction for multiple comparisons showed there to be a significant difference between 1 and 13${}^{\circ }$/s, (*p* = 0.042), between 4 and 13${}^{\circ }$/s (*p* = 0.015), none of the other comparisons were statistically significant.

### Appendix C.2. Threshold Elevation for Motion Against Parallel Backgrounds

For motion against parallel backgrounds, there is a significant main effect of speed (F(3.07,144.38) = 5.43, *p* = 0.001, $\eta_{G}^{2}$ = 0.08), and a significant interaction between migraine group and speed (F(3.07,144.38) = 4.46, *p* = 0.005, $\eta_{G}^{2}$ = 0.07). Post-hoc tests showed there to be a significant difference between control and migraine groups at 1 ${}^{\circ }$/s (*p* = 0.030), and at 13${}^{\circ }$/s (*p* = 0.0076), none of the other comparisons were statistically significant when adjusted for multiple comparisons using the Bonferroni correction.

### Appendix C.3. Difference in Threshold Elevation between Orthogonal and Parallel Backgrounds

There was a significant main effect of group on the difference in threshold elevation between orthogonal and parallel motion (F(1,47) = 4.82, *p* = 0.03, $\eta_{G}^{2}$ = 0.03). There was also a significant main effect of speed (F(3.13,147.02) = 33.63, *p* = < 0.0001, $\eta_{G}^{2}$ = 0.34). There was a significant interaction between migraine group and speed (F(3.13,147.02) = 4.06, *p* = 0.007, $\eta_{G}^{2}$ = 0.06). There was a significant difference between the migraine and control group at 13 ${}^{\circ }$/s (*p* < 0.0001), none of the other comparisons were statistically significant when corrected for multiple comparisons using the Bonferroni correction.

### Appendix C.4. Relationship to Headache Frequency

A general linear mixed effects model was created to predict the difference in threshold elevation between orthogonal and parallel backgrounds, including headache frequency and speed as a fixed factors, and subject as a random factor. This was compared to a null model that included only speed and subject as factors. There was a relationship between headache frequency and the difference in threshold elevation ($\chi^{2}$ (1) = 4.23, *p* = 0.040). This increased the difference in threshold elevation by 0.23dB (0.11 standard error).
