# Supplementary material for: Temporal Integration of Motion Streaks in Migraine
Source: Vision (Basel). 2018 Jul 13;2(3):27. doi: 10.3390/vision2030027 (PMC6836222; doi:10.3390/vision2030027)
Supplement: Supplementary file 1 [file vision-02-00027-s001.zip › supplementary information/Supplementary information.docx]

Supplementary information notes

The raw data for experiment 1 (see Appendix) in folder "results". This includes a folder for each observer. Inside this is a .txt file containing their responses for the experiment. Columns represent: trial number, dot speed, dot size, bandwidth, background orientation, contrast level, direction (left or right), response (correct or incorrect), reaction time (ms).

The raw data for experiment 2 (see Appendix) in folder "resultsmark2". This includes a folder for each observer. Inside this is a .txt file containing their responses for the experiment. Columns represent: trial number, dot speed, dot size, bandwidth, background orientation, contrast level, direction of motion (horizontal or vertical), direction (left or right for horizontal motion, up or down for vertical motion), response (correct or incorrect), reaction time (ms).

The raw data for experiment 3 (main experiment, see manuscript) in folder "resultsmark3". This includes a folder for each observer. Inside this is a .txt file containing their responses for the experiment. Columns represent: trial number, dot speed, dot size, bandwidth, background orientation, contrast level, direction of motion (horizontal or vertical), direction (left or right for horizontal motion, up or down for vertical motion), response (correct or incorrect), reaction time (ms).

XLS files contain the migraine questionnaires relating to each of the three parts of the experiment, "migraineQUStreakAppendixExperiment1.xls" contains headache questionnaire information for Experiment 1 (Appendix), "migraineQUStreakAppendixExperiment2.xls" contains headache questionnaire information for Experiment 2 (Appendix), "migraineQUmainExperiment.xls" contains headache questionnaire, results for the FrACT test, and Pattern Glare Test for the main experiment. Individuals included in the final analysis are coloured, red indicates the migraine group. Blue indicates the control group. In the migraineQUmainExperiment file, participant numbers 8, 11 and 12 did not meet the IHS criteria, and so were excluded, however these participants to have a diagnosis of migraine by a medical professional and so were included in the analysis in the Appendix. Participant 8 was excluded due to a diagnosis of vestibular migraine, participant 19 was excluded due to a diagnosis of cluster headache. Participant 20 did not meet the IHS criteria. Participant 26 had a headache only 1 day ago, and so was excluded. Participant 29 did not meet the IHS criteria and so was excluded, participant 30 had a diagnosis of tension headache. Of note, participant 15 met the IHS criteria, and so was included, but this individual has a diagnosis of abdominal migraine. For the analysis in the analysis in the appendix (those with a diagnosis of migraine by a medical professiona), participant numbers of those included are as follows: participants 1, 3, 4, 5, 6, 7, 8, 10, 11, 12, 13, 14, 15, 16, 17, 21, 22, 24, 25, 28. Participant 8 does not meet the IHS criteria for migraine, but has a medical professional diagnosis of vestibular migraine. Participant 11 and 12 also do not meet the IHS criteria for migraine, but have a diagnosis from a medical professional. In both of these cases, the individuals were not included in the analysis in the main body of the text (as they did not meet the IHS criteria), but they do have a medical professional diagnosis of migraine, and so were included in this analysis.
